# Inhibition of nuclear receptor RORα attenuates cartilage damage in osteoarthritis by modulating IL-6/STAT3 pathway

**DOI:** 10.1038/s41419-021-04170-0

**Published:** 2021-09-28

**Authors:** Tongzhou Liang, Taiqiu Chen, Jincheng Qiu, Wenjie Gao, Xianjian Qiu, Yuanxin Zhu, Xudong Wang, Yanbo Chen, Hang Zhou, Zhihuai Deng, Pengfei Li, Caixia Xu, Yan Peng, Anjing Liang, Peiqiang Su, Bo Gao, Dongsheng Huang

**Affiliations:** 1 Department of Orthopedics, Sun Yat-sen Memorial Hospital of Sun Yat-sen University, Guangzhou, Guangdong China; 2 Department of Orthopedics, The First Affiliated Hospital of Sun Yat-sen University, Guangzhou, Guangdong China; 3 Research Centre for Translational Medicine, First Affiliated Hospital, Sun Yat-sen University, Guangzhou, Guangdong China; 4 Guangdong Provincial Key Laboratory of Orthopedics and Traumatology, First Affiliated Hospital, Sun Yat-sen University, Guangzhou, Guangdong China

**Keywords:** Nuclear receptors, Osteoarthritis

## Abstract

Osteoarthritis (OA) is characterized by cartilage destruction, chronic inflammation, and local pain. Evidence showed that retinoic acid receptor-related orphan receptor-α (RORα) is crucial in cartilage development and OA pathogenesis. Here, we investigated the role and molecular mechanism of RORα, an important member of the nuclear receptor family, in regulating the development of OA pathologic features. Investigation into clinical cartilage specimens showed that RORα expression level is positively correlated with the severity of OA and cartilage damage. In an in vivo OA model induced by anterior crucial ligament transaction, intra-articular injection of si-Rora adenovirus reversed the cartilage damage. The expression of cartilage matrix components type II collagen and aggrecan were elevated upon RORα blockade. RNA-seq data suggested that the IL-6/STAT3 pathway is significantly downregulated, manifesting the reduced expression level of both IL-6 and phosphorylated STAT3. RORα exerted its effect on IL-6/STAT3 signaling in two different ways, including interaction with STAT3 and IL-6 promoter. Taken together, our findings indicated the pivotal role of the RORα/IL-6/STAT3 axis in OA progression and confirmed that RORα blockade improved the matrix catabolism in OA chondrocytes. These results may provide a potential treatment target in OA therapy.

## Introduction

Osteoarthritis (OA) is one of the most common degenerative orthopedic diseases, affecting 250 million people worldwide [1]. However, due to its unclear etiology, the prediction and early intervention of OA are difficult. The most significant features in degenerated cartilage are the loss of matrix components (type II collagen, aggrecan) and the accumulation of matrix degradation enzymes such as metalloproteinase (MMP) and a disintegrin and metalloproteinase with thrombospondin motifs (ADAMTS) [2, 3]. However, once these pathological features occur, the progression of cartilage damage is usually irreversible. In the later stage of OA, patients may develop joint stiffness, local pain, and limited mobility, which eventually require total knee replacement (TKA) surgery. Hence, in-depth investigation into the common upstream pathogenic factor may contribute to advancements in therapeutic targets for OA.

Orphan nuclear receptors (ONRs), originally named because their endogenous ligands were yet to be found, consist of a complex transcriptional network that regulates cellular activity in different cell types [4]. The retinoic acid receptor-related orphan receptor-α (RORα) is an important member of the ONR family. Previous studies have shown that RORα is a relatively evolutionarily conserved protein and participates in multiple biological functions, including lipid metabolism, inflammation, and circadian rhythm [5–7]. Moreover, recent studies indicate that RORα is crucial in osteochondral development and degeneration. The staggerer (sg) mice carry spontaneous *Rora* gene mutants that cause subsequent RORα deletion. The homozygous (sg/sg) mice exhibit cerebellar ataxia, bone mineral content loss, and osteopenic phenotype [8, 9]. Our studies suggested RORα regulates nucleus pulposus matrix metabolism and apoptosis [10]. Moreover, RORα contributes to chondrocyte hypertrophy during the development of endochondral bone and growth plates, acting as a prehypertrophic marker [11, 12]. Further, a recent study indicates that the cholesterol metabolism pathway is involved in OA pathogenesis, and RORα is a critical regulator of cholesterol metabolism [13]. Despite these studies, the mechanisms and pathways by which RORα elicits its effects in cartilage and whether RORα can serve as a therapeutic target remain poorly understood.

Here, we report that RORα blockade protects against OA development by promoting matrix anabolism and preventing catabolism. Considering the intimate relationship between RORα, cytokines production, and inflammatory response, we sought to determine which pathway is affected by RORα in chondrocytes. In this study, we identified the IL-6/STAT3 pathway was significantly downregulated upon RORα blockade. RORα blockade significantly reversed the damage in human chondrocytes caused by IL-6 treatment. We further investigated the molecular mechanism by which RORα blockade regulates IL-6/STAT3 in chondrocytes. Our results indicated the potential therapeutic implications of RORα in treating cartilage defects and OA.

## Results

### Increased expression of RORα in articular cartilage of OA patients and surgically induced OA mice

**Fig. 1:**
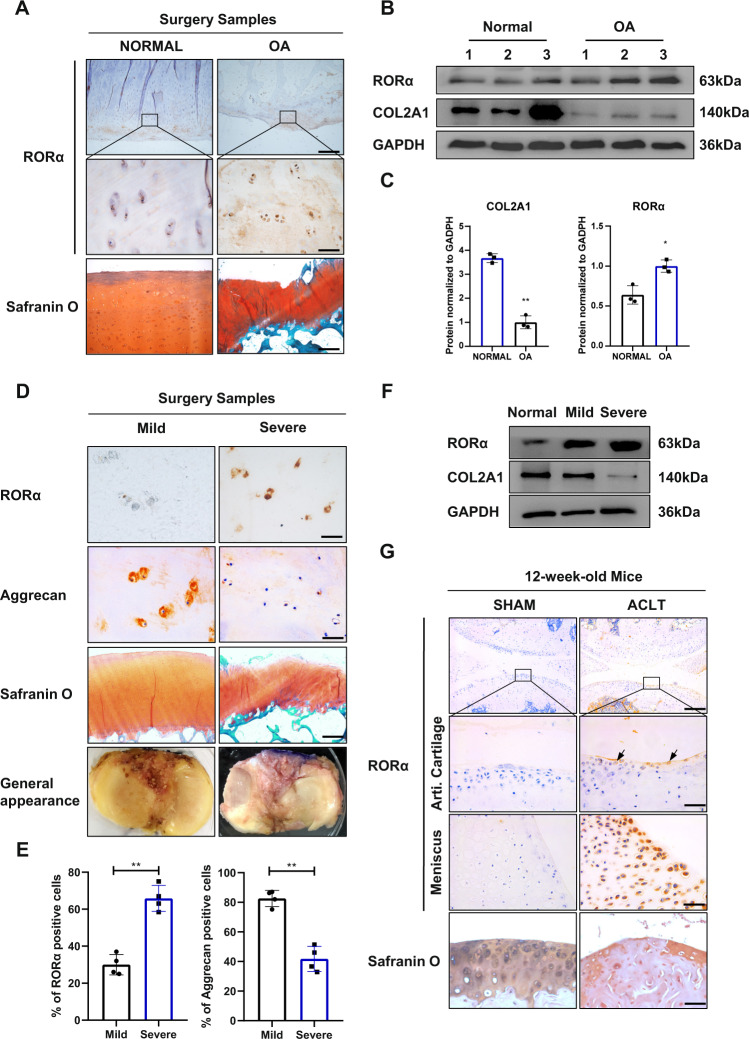
The expression level of RORα is positively correlated with the severity of OA and cartilage damage in human OA samples and surgery-induced OA mice model. **A** IHC and safranin O staining for detection of RORα in OA samples and controls. Boxed areas indicate the typical area of RORα expression. Scale bars, 500 and 50 μm for ×40 and ×400 images, respectively. **B** Representative western blot images for detection of RORα and COL2A1 in the cartilage tissue from control and OA patients (*n* = 3). **C** The quantification of western blot for detection of RORα and COL2A1 from OA patients and normal control. **D** Representative images of IHC staining for RORα and aggrecan, Safranin O staining, and the general appearance of different severity of OA samples. Scale bars, 50 μm for IHC staining and 500 μm for Safranin O staining images. **E** The percentage of RORα-positive cells and aggrecan-positive cells in mild or severe degenerated cartilage samples from OA patients (*n* = 4). **F** Western blot for detection of RORα and COL2A1 from the cartilage tissue of different severity of OA. **G** IHC and safranin O staining for RORα in mouse knee joints articular cartilage and meniscus 8 weeks after OA mouse model creation via ACLT surgery (*n* = 3). Scale bars, 200 and 50 μm for the upper and the middle images, respectively. The statistical data in **C** and **E** were analyzed with Student’s *t* test. *P < 0.05; **P < 0.01. All data shown above are presented as the mean ± SD.

Using cartilage samples collected from amputation or tumor resection surgery as normal control and TKA surgery as OA cartilage, we performed immunohistochemistry (IHC) staining to detect RORα expression level. The expression level of RORα was significantly elevated in human OA cartilage samples compared with normal cartilage, and the high level of RORα protein is positively associated with cartilage damage (Fig. 1A). This result was confirmed by western blotting (Fig. 1B, C). We further divided the patients into three grades of degeneration based on plain radiography findings and the general appearance of the specimen. The specimens were divided into severe and mild degeneration categories according to the Kellgren-Lawrence (KL) scoring and modified Outerbridge classification [14]. Safranin O staining showed that the thickness of articular cartilage was impaired in severely degenerated samples. Moreover, the expression of RORα was increased significantly in the superficial zone of severely degenerated cartilage, whereas the expression of aggrecan decreased (Fig. 1D). A marked increase of RORα-positive cells was observed in severely degenerated cartilage samples (Fig. 1E). This finding was confirmed by Western blotting (Fig. 1F). Next, we constructed an ACLT-induced OA mice model to verify the expression of RORα in mice with surgery-induced OA. The mice were sacrificed 8 weeks after ACLT or sham surgery. Compared with the mice in the sham-surgery group, a significant elevation in RORα expression was observed in the articular cartilage of OA mice. Furthermore, consistent with the finding in human specimens, RORα is especially abundant in the superficial layer of the articular cartilage, indicating that its presence may be associated with cartilage erosion (Fig. 1G). Taken together, these results demonstrated that RORα was upregulated in both mouse and human damaged cartilage samples in OA and might contributes to the development of OA.

### RORα knockdown attenuated cartilage damage in OA mouse model

**Fig. 2:**
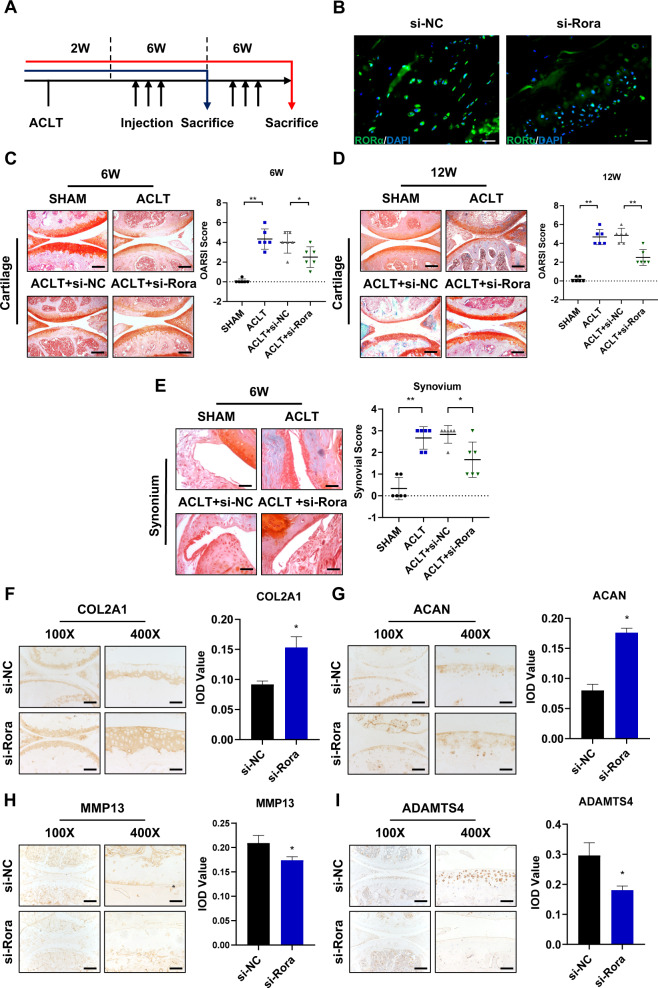
RORα knockdown alleviated surgery-induced osteoarthritis in mice. **A** Experimental scheme. A total number of 48 female, 12-week-old mice were used in the experiment. Two weeks after ACLT surgery, intra-articular si-Rora or scrambled (negative control) siRNA injection was performed after 6 or 12 weeks. *n* = 12 for each group, half of the mice (*n* = 6) were killed at 6 weeks. **B** The immunofluorescence image of RORα (green) and DAPI (blue) 5 days after intra-articular injection of si-Rora or control siRNA (si-NC). Scale bars, 50 μm. **C** Representative images of safranin O staining and OARSI scoring of the articular cartilage 6 weeks after injection (*n* = 6). Scale bars, 200 μm. **D** Representative images of safranin O staining and OARSI score of the articular cartilage 12 weeks after injection (*n* = 6). Scale bars, 200 μm. **E** Safranin O staining of the synovium and synovial score of mice treated with indicated treatments as shown after 12 weeks after OA surgery (*n* = 6). Scale bars, 200 μm. **F**–**I** IHC staining of representative paraffin sections of COL2A1 (**F**), Aggrecan (**G**), MMP13 (**H**), ADAMTS4 (**I**), and their integrated optical density (IOD) scoring respectively. Scale bars, 200 μm for the ×100 image and 50 μm for the ×400 image. The statistical data in **C**–**E** were analyzed with Mann–Whitney *U* test, and data in **F**–**I** were analyzed with Student’s *t* test. *P < 0.05, **P < 0.01. All data shown above are presented as the mean ± SD.

The effect of RORα blockade was then assessed in the ACLT-induced OA mouse model. RORα*-*specific knockdown in mice was achieved via intra-articular injection of si-Rora adenoviral particles. Two weeks after ACLT surgery, the adenoviral particles of either si-Rora or control siRNA (si-NC) were injected every 7 days. After 6 weeks of treatment, half of the mice were killed, and articular cartilage samples were subjected to histological examination. The remaining mice continued treatment for 6 weeks and were killed at 12 weeks after initial injection (Fig. 2A). The infection and knockdown efficiency was measured after adenovirus injection by immunofluorescence staining of RORα (Fig. 2B and Supplementary Fig. 1A). Histomorphometric features were assessed via safranin O staining and the Osteoarthritis Research Society International (OARSI) scoring system. In line with our expectations, whereas the articular cartilage of the sham-operated mice remained relatively intact, si-Rora injection partially reversed the progression of cartilage damage compared to saline or si-NC injection (Fig. 2C). A similar pattern was observed 12 weeks after injection (Fig. 2D). The severity of synovium inflammation was also alleviated upon si-Rora injection (Fig. 2E). Significant loss of extracellular components was observed in both ACLT and si-NC groups. However, si-Rora injection restored aggrecan and COL2A1 expression in the cartilage (Fig. 2F, G). Furthermore, the expression of MMP13 and ADAMTS4 was decreased in the si-Rora injection group (Fig. 2H, I). Taken together, the result suggested the RORα blockade promotes chondrocyte anabolism and suppresses chondrocyte catabolism and, therefore, alleviating OA progression in vivo.

### RORα regulated the catabolism and anabolism of chondrocyte

**Fig. 3:**
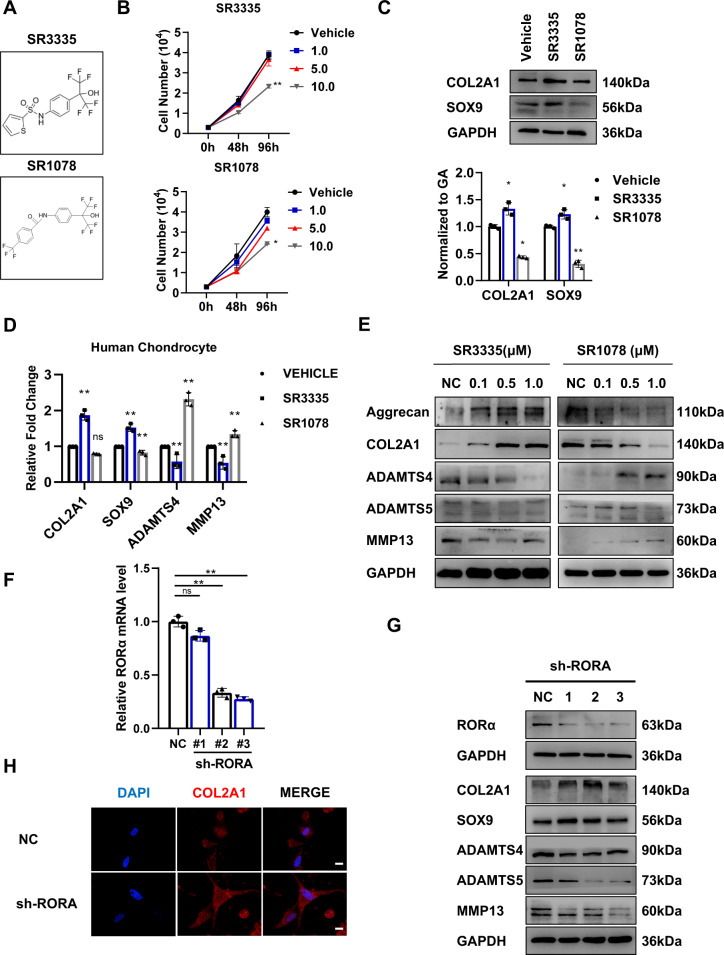
Manipulation of RORα via small molecular regulator and siRNA regulated chondrocyte metabolism. **A** Chemical structures of RORα antagonist SR3335 and agonist SR1078. **B** Primary chondrocytes were treated with different concentrations of SR3335 and SR1078 (0, 1, 5, 10 μM). After the indicated time points, viable cells were counted using CCK-8 assay. **C** Western blot analysis and quantification of COL2A1 and SOX9 expression, which are regulated by when treated with 1 μM SR3335 and SR1078 for 48 h. **D** The chondrocytes were treated with vehicle, 1 μM SR1078, or 1 μM SR3335 for 48 h. The mRNA level of *COL2A1*, *SOX9*, *ADAMTS4*, and *MMP13* was determined with real-time PCR. **E** Western blot analysis of the indicated proteins in human chondrocytes treated with vehicle or the indicated concentration of compounds for 72 h. **F** The relative expression of RORA gene after infected with sh-RORA or sh-NC lentivirus for 72 h. **G**, **H** Western blot analysis and representative immunofluorescence images of indicated protein after sh-NC and sh-RORA infection. Scale bars, 20 μm. Representative blots and images (*n* = 3). The statistical data in **B** were analyzed with one-way ANOVA, data in **D** and **F** were analyzed with Student’s *t* test. *P < 0.05, **P < 0.01, ns = no significance. All data shown above are presented as mean ± SD.

We then examined the in vitro effect of RORα blockade in human chondrocytes. Primary chondrocytes used in this section were isolated from three OA patients and experiments were performed in different chondrocyte lines as biological replicates. To explore the effect of RORα in regulating chondrocytes extracellular matrix (ECM) production and degradation, we employed two well-established small-molecule regulators of RORα, antagonist SR3335, and agonist SR1078 (Fig. 3A). The binding of these ligands to the ligand-binding domain leads to conformational transformation and subsequent functional alternations. Compared with vehicle-treated chondrocytes, SR1078 and SR3335 treatment did not affect cell viability at a concentration below 5 μM (Fig. 3B). We found that the protein level of COL2A1 and SOX9 were elevated in human chondrocytes treated with SR3335, whereas SR1078 treatment led to a decrease in COL2A1 and SOX9 expression (Fig. 3C). As expected, the mRNA level of cartilage matrix component COL2A1 and cartilage marker SOX9 elevated. In contrast, the expression level of ADAMTS4 and MMP13 was suppressed upon SR3335 treatment. SR1078 exerted an opposite effect on chondrocytes, promoting the expression of MMP13, ADAMTS4, and ADAMTS5, whereas inhibiting COL2A1 and SOX9 (Fig. 3D). We further investigated whether RORα agonist and antagonist regulated the expression of these proteins in a dose-dependent manner and sought to determine the optimal doses of treatment. Consistently, SR3335 treatment resulted in a downregulation of the proteins ADAMTS4, ADAMTS5, and MMP13, whereas aggrecan and COL2A1 were upregulated significantly (Fig. 3E). This effect was dose-dependent and reached a plateau at a concentration of 0.5–1 μM, suggesting that 0.5 or 1 μM is an optimal concentration for treating chondrocytes. In addition to transcription modulation of RORα, we further investigated the effect of RORα blockade by sh-RORA lentivirus. The infection and knockdown efficacy were confirmed (Fig. 3F and Supplementary Fig. 1B). Similarly, RORA knockdown predominantly downregulated MMP13 and ADAMTS4, whereas the expressions COL2A1 and SOX9 upregulated (Fig. 3G, H). Therefore, these data demonstrated that RORα blockade inhibits cartilage ECM catabolism and promotes anabolism of ECM while exerting a little effect on proliferation.

### RORα blockade downregulated the IL-6 response pathway

**Fig. 4:**
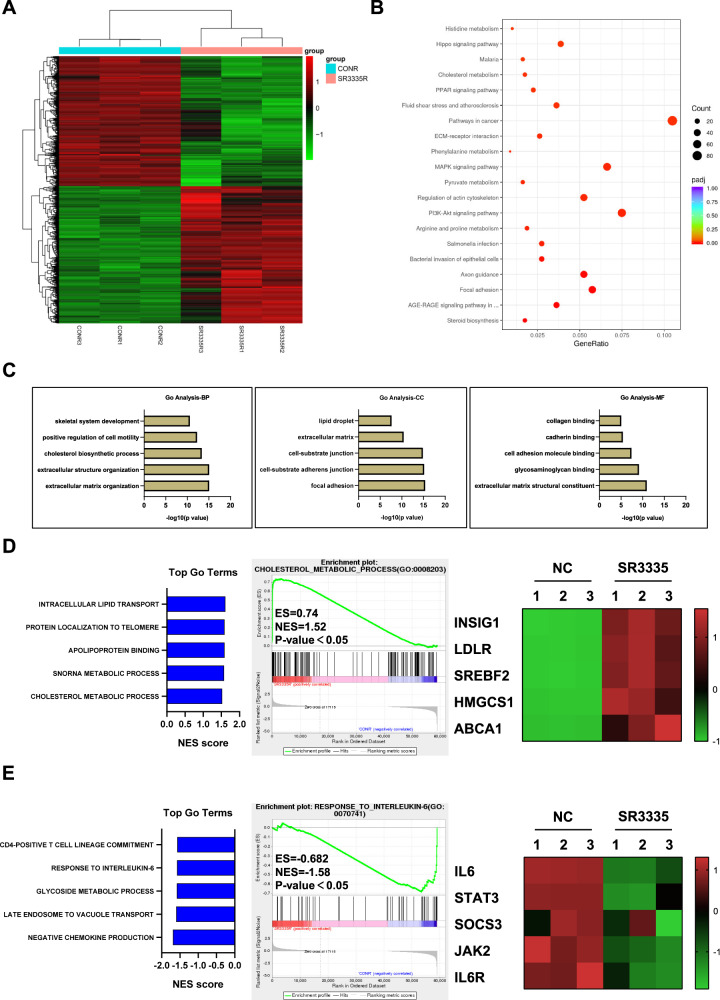
RORα affected the IL-6-related pathways. **A** The heatmap of DEGs, as detected by RNA-seq in human articular chondrocytes treated with either SR3335 (1 µM) or vehicle (DMSO) for 48 h (*n* = 3 biological replicates). **B** KEGG and **C** GO terms with the most significant *P* values. *BP* biological process, *CC* cellular component, *MF* molecular function. **D** GSEA of positively associated GO terms with SR3335 treatment (left panel) and cholesterol metabolic hallmark genes in chondrocytes treated with RORα antagonist compared with vehicle (middle and right panel). **E** GSEA of negatively associated GO terms with SR3335 treatment (left panel) and IL-6-associated genes in chondrocytes treated with SR3335 compared with vehicle (middle and right panel).

To identify the key factors in OA pathogenesis that RORα regulates, RNA-seq was employed in human articular chondrocytes obtained from three OA patients after 1 μM SR3335 treatment for 48 hours. A total of 1852 protein-encoding differentially expressed genes (DEGs) were identified (Fig. 4A). Among these DEGs, 902 DEGs were downregulated and 950 DEGs were upregulated. Notably, RORα blockade in chondrocytes upregulated the expression of genes that are known to participate in cartilage development and homeostasis, including *ACAN*, *COL2A1*, *CCN1*, and *CCN2* (Supplementary Fig. 2A). Although genes associated with cartilage destruction (*COMP*), fibroblast-like markers (*COL3A1*, *COL1A2*), and matrix degradation enzymes (*ADAMTS4*, *MMP13*) were downregulated [15, 16]. Kyoto Encyclopedia of Genes and Genomes (KEGG) pathway analysis identified several pathways that are associated with RORα, including the AGE-RAGE signaling pathway, PPAR signaling pathway, and Hippo pathway (Fig. 4B). GO analysis revealed that ECM organization, cholesterol biosynthetic process, and skeletal system development are among the top five most affected biological processes upon RORα blockade (Fig. 4C). Further examination by gene-set enrichment analysis (GSEA) suggested that the cholesterol metabolic process was strongly upregulated by SR3335 treatment. The expression of cholesterol transportation genes, such as *INSIG1*, *LDLR*, and *SREBF2*, were also upregulated in the SR3335-treated group (Fig. 4D). Besides, hallmarks of the IL-6 response pathway were significantly downregulated (Fig. 4E). IL-6 response pathway included several kinases and factors that participated in signal transduction, including Janus Kinase (JAK) and signal transducer and activator of transcription (STAT). The result of RNA-seq confirmed that RORα regulated different biological processes in chondrocytes, including cartilage ECM homeostasis, cholesterol metabolism, and inflammation response.

### RORα blockade alleviated IL-6-induced chondrocyte degeneration

**Fig. 5:**
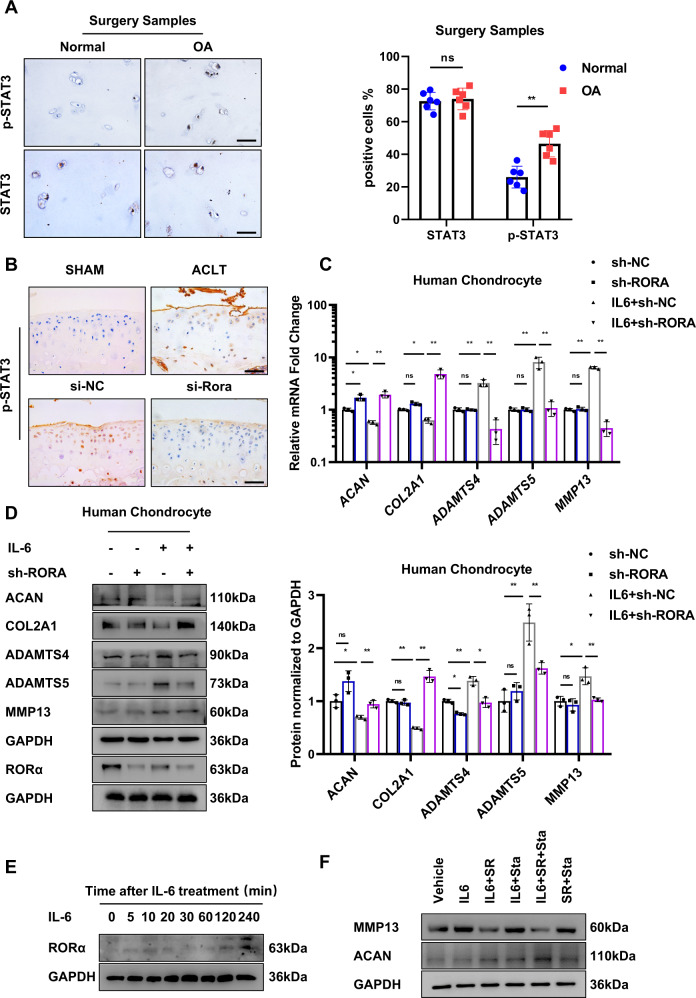
RORα inhibition reversed IL-6 mediated cartilage degradation in human articular chondrocytes. **A** IHC staining and positive cell percentage for STAT3 and phosphorylated STAT3 (Y705) of normal and degenerated OA cartilage (*n* = 6). Scale bars, 50 μm. **B** IHC staining for phosphorylated STAT3 of articular cartilage after 6 weeks of indicated treatment of mice. Scale bars, 50 μm. **C** Cells were infected with sh-RORA or control virus (sh-NC) lentivirus and then treated with 50 ng/ml IL-6 for 48 h. The expressions of *MMP13*, *ADAMTS4*, *ADAMTS5*, *ACAN*, and *COL2A1* in human articular chondrocytes were detected by real-time PCR analysis. **D** Western blot analysis and quantification of ACAN, COL2A1, ADAMTS4, ADAMTS5, and MMP13 protein level in human chondrocytes. Chondrocytes were infected with sh-RORA sh-NC lentivirus and then treated with 50 ng/ml IL-6 for 48 h as indicated. **E** The expression of RORα in human articular chondrocytes after different durations of IL-6 treatment was detected by western blot. **F** The expressions of MMP13, aggrecan, and COL2A1 in human chondrocytes were detected by western blot. Cells were treated with either vehicle, IL-6, SR3335, Stattic, or in combination as indicated above. The statistical data in **A** were analyzed with Student’s *t* test, and data in **C** and **D** were analyzed with one-way ANOVA followed by Dunnett’s test. *P < 0.05, **P < 0.01, ns = no significance. All data shown above are presented as mean ± SD.

To investigate the function of IL-6/STAT3 signaling pathway in OA pathogenesis, we explored whether the phosphorylation state of the key factor of the IL-6 response pathway, STAT3, was altered in OA patients. The expression of STAT3 total protein remained relatively unchanged, whereas the expression of phosphorylated STAT3 (Tyr705) increased progressively with OA severity in patients (Fig. 5A). The result is consistent with previous research, which showed IL-6/STAT3 pathway activation is positively associated with cartilage damage [17]. Next, we examined whether the expression of phosphorylated STAT3 is affected in vivo. IA injection of si-Rora adenovirus attenuated the elevated p-STAT3 expression in the cartilage of OA mice (Fig. 5B). Moreover, to explore whether RORα blockade reversed the hostile effect of IL-6, we treated cultured human chondrocytes isolated from three normal control patients with exogenous IL-6. The expression level of ADAMTS4, ADAMTS5, and MMP13 was elevated upon IL-6 stimulation, whereas the expression of ACAN and COL2A1 was downregulated. However, RORα knockdown in chondrocytes restored the dysregulation of these protein levels, suggesting RORα restored the IL-6-induced chondrocyte degeneration (Fig. 5C, D). Next, we examined whether the expression of RORα was altered by IL-6 treatment. We found that the level of RORα was sightly induced upon IL-6 treatment in a long duration of treatment (Fig. 5E). To examine whether inhibiting RORα and the downstream effector STAT3 simultaneously yield a greater extent of inhibiting matrix degradation, we applied Stattic, a well-established and high-selective STAT3 inhibitor on chondrocytes. The expression of MMP13 was significantly downregulated in both Sttatic and SR3335-treated groups compared with Sttatic or SR3335-treated alone (Fig. 5F). Together, these results suggest that IL-6/STAT3 signaling pathway are involved in OA pathogenesis and RORα blockade partially ameliorate the chondrocytes dysfunctions.

### RORα attenuated STAT3 phosphorylation via interacting with STAT3 and regulating IL-6 expression

**Fig. 6:**
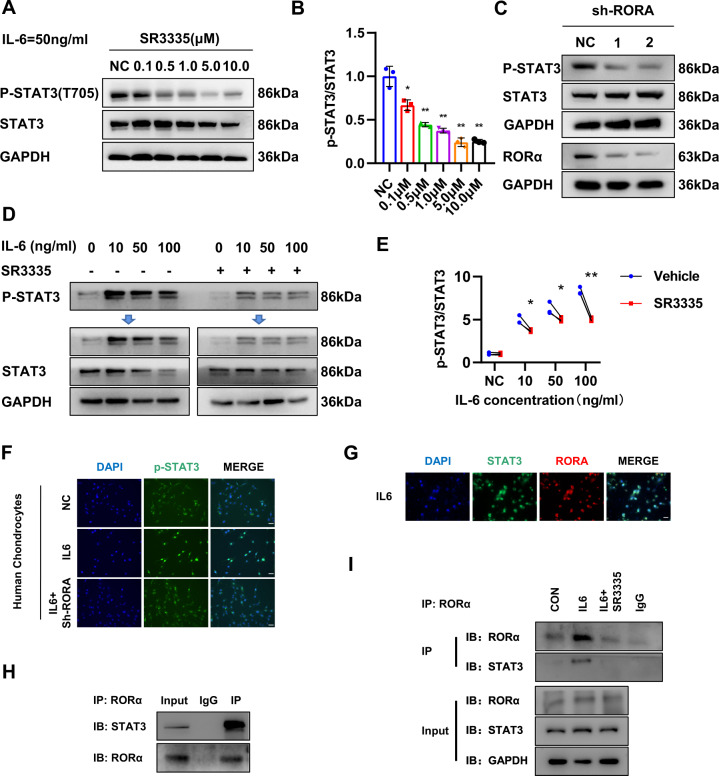
RORα regulates STAT3 phosphorylation via binding with STAT3. **A**, **B** The expression of STAT3 and phosphorylated STAT3 (Y705) in human articular chondrocytes after pretreated with a gradient concentration of SR3335 for 6 h and a 50 ng/ml IL-6 treatment for 30 min was detected by Western blot. Quantification was conducted and the ratio of p-STAT3/STAT3 was calculated. **C** The expression of STAT3 and phosphorylated STAT3 (Y705) in human articular chondrocytes after sh-RORA infection for 72 h and IL-6 treatment for 30 min were detected by western blot. **D**, **E** The expression level and quantification of phosphorylated STAT3 (Y705) in isolated human articular chondrocytes after pretreated with 1 μM SR3335 for 6 h and then treated with a gradient concentration of IL-6 for 30 min. **F** The chondrocytes were infected with sh-RORA or sh-NC lentivirus for 72 h and treated with IL-6 (50 ng/ml) for 30 min. Slices were harvested after indicated treatment and subjected to fluorescence microscopy of p-STAT3/DAPI staining in formaldehyde-fixed cells. Scale bar = 50 μm. **G** Slices of human chondrocytes were harvested after indicated treatment and subjected to fluorescence microscopy of RORα, STAT3, and DAPI staining in formaldehyde-fixed cells. Scale bar = 50 μm. **H** Immunoprecipitation was performed on chondrocytes after IL-6 treatment for 1 h with anti-RORα antibody. **I** Immunoprecipitation was performed with anti-RORα antibody after treatment with vehicle, 50 ng/ml IL-6, or 50 ng/ml IL-6, and 1 μM SR3335 for 1 h. Representative blots and images (*n* = 3). The statistical data in **E** were analyzed with Student’s *t* test, and data in **B** were analyzed with one-way ANOVA followed by Dunnett’s test. *P < 0.05, **P < 0.01, ns = no significance. All data shown above are presented as mean ± SD.

Forming STAT3 dimers after phosphorylation is the major mechanism that STAT3 regulates downstream gene expression and cellular biological processes [18]. The phosphorylation of STAT3 is controlled by the interaction between IL-6 and its receptor gp130 with the assistance of JAK. Therefore, we investigated whether RORα blockade affects the phosphorylation state of STAT3. In chondrocytes, RORα blockade by SR3335 prevented the phosphorylation of STAT3 under IL-6 treatment in a dose-dependent manner (Fig. 6A, B). Besides, RORα knockdown in chondrocytes decreased the phosphorylation of STAT3 (Fig. 6C). Then we explored the phosphorylation status of STAT3 under different concentrations of IL-6 and found that RORα blockade blunted STAT3 phosphorylation under IL-6 stimulation. In contrast, the expression of phosphorylated STAT3 was higher without SR3335 treatment (Fig. 6D, E). Furthermore, immunofluorescence analysis revealed that STAT3 was imported to the nucleus after IL-6 stimulation that was abolished upon SR3335 treatment (Fig. 6F). To elucidate how RORα regulated IL-6/STAT3 pathway, we tried to find out whether RORα interacts with STAT3 via direct binding. We observed colocalization of RORα and STAT3 expression under IL-6 treatment (Fig. 6G). Endogenous immunoprecipitation suggested a possible physical interaction between STAT3 and RORα (Fig. 6H). Next, we evaluated whether the binding of RORα with STAT3 is affected by IL-6 stimulation. Further study suggested that the binding increased under IL-6 treatment and was suppressed under SR3335 treatment (Fig. 6I). These findings demonstrated that RORα affected the phosphorylation of STAT3 through direct interaction with STAT3 protein.

**Fig. 7:**
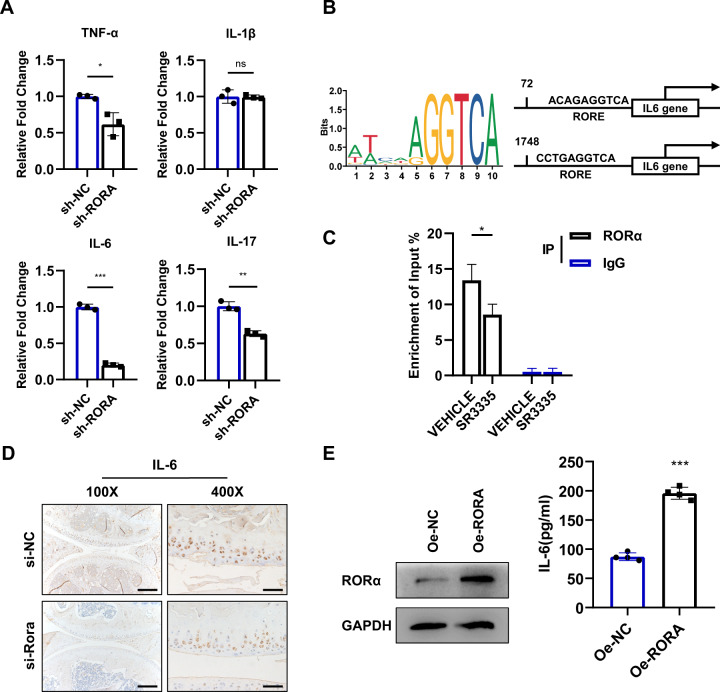
RORα regulates IL-6 expression by interacting with IL-6 promoters. **A** Real-time PCR analysis of TNF-α, IL-1β, IL-6, and IL-17 mRNA in human chondrocytes infected with RORA knockdown lentivirus (sh-RORA) or control lentivirus (sh-NC) (*n* = 3). **B** Schematic diagram of the potential binding site for RORα in the promoter region of IL-6 using JASPAR database. **C** ChIP-qPCR enrichment assay was performed on chondrocytes with anti-RORα antibody or IgG. The chondrocytes were pretreated with vehicle or 1 μM SR3335 for 6 h. IgG immunoprecipitation was used as a negative control. **D** IHC staining for IL-6 of articular cartilage after indicated adenovirus injection for 6 weeks in mice. Scale bars, 200 μm for ×100 picture and 50 μm for ×400 picture. **E** ELISA assay to detect IL-6 protein expression after transfected with RORA overexpression or vector plasmid for 48 h (*n* = 4). The statistical data in **A**, **C**, and **E** were analyzed with Student’s *t* test. *P < 0.05, **P < 0.01, ***P < 0.001, ns = no significance. All data shown above are presented as mean ± SD.

Next, the expression level of several pro-inflammatory cytokines was measured. The mRNA expression of TNF-α, IL-17, and IL-6 was suppressed upon RORA knockdown (Fig. 7A). JASPAR analysis indicated that RORα may control its target gene transcription by binding to RORE sites, and the *IL6* gene possesses possible RORE sites (Fig. 7B). CHIP-qPCR assay was applied to confirm RORα-binding sites on *IL-6* promoter region (Fig. 7C). IA injection of si-Rora adenovirus for 6 weeks inhibited the IL-6 production in articular cartilage in vivo (Fig. 7D). To further confirm the effect of RORα on IL-6 in vitro, we overexpressed RORα in human chondrocytes and the cell cultural supernatant was collected. ELISA assay confirmed the level of IL-6 was upregulated upon RORα and STAT3 overexpression, suggesting that STAT3 and RORα both contributed to the upregulated level of IL-6 (Fig. 7E, Supplementary Fig. 3). Taken together, these results confirmed that RORα is deeply involved in IL-6/STAT3 signaling pathway by interacting with STAT3 protein or regulating the *IL6* gene transcriptional activity in chondrocytes.

## Discussion

OA has become an increasing burden in an aging society. Identifying the key factors regulating cartilage matrix anabolism and catabolism is an indispensable process in developing disease-modifying osteoarthritis drug. Our study provided evidence for the potential role of RORα, a member of the nuclear receptor family, in OA pathogenesis. RORα expression increased significantly in OA patients and correlated positively with OA severity. In addition, we found that RORα blockade reversed the cartilage damage and synovium inflammation of OA mice model. Expression of aggrecan of mice articular cartilage in the RORα inhibition group increased, and the levels of MMP13 decreased. In vitro studies suggested that RORα blockade promotes matrix anabolism and inhibits matrix catabolism in human chondrocytes. In addition, RORα blockade suppressed the IL-6/STAT3 pathway, a key pathway in OA pathogenesis. We also investigated in which way RORα regulates IL-6/STAT3 signaling pathway and identified direct binding with STAT3 or interaction with *IL6* promoter as a possible mechanism.

Nuclear receptors, such as ERRγ, PPARγ, and NR1D1, have been implicated to be involved in OA pathogenesis [19–21]. Moreover, GWAS studies suggest that certain single-nucleotide polymorphism of the nuclear receptor coactivator 3 increased patient susceptivity to hip OA and resulted in the disruption of chondrocyte metabolism [22, 23]. Therefore, the nuclear receptor family is deeply involved in the pathogenesis of OA to a complex extent, but the underlying mechanisms are yet to be elucidated [24]. RORα was first discovered to be involved in the onset of cerebellar ataxia [25, 26]. The possible ligand of RORα has been searched for many years. Cholesterol, lipid metabolites, and melatonin have been found to bind with RORα and regulate its transcription activity [27–29]. Moreover, RORα can also regulate the transcriptional activity of the target gene in a ligand-independent way [30]. In this research, we found that RORα blockade by IA injection reversed the cartilage damage by regulating the expression of matrix components and degrading enzymes. However, this study did not reveal the role of the above-mentioned RORα endogenous ligands in modulating OA pathogenesis. Further studies should address the clinical relevance between these ligands and OA severity.

IL-6 is a member of the chemokines family and has a central role in inflammation [31]. Increased IL-6 serum level correlates with disease incidence and the severity of knee OA [32]. Many extracellular factors, such as cytokines and mineral crystals, are found to stimulate the secretion of IL-6 [33, 34]. IL-6 binds to gp130 receptor complex and activates JAKs, and subsequently phosphorylates and activates STAT3 [35, 36]. In the skeletomuscular system, IL-6/STAT3 pathway is crucial in multiple biological processes like chondrogenesis and ossification [34, 37]. Further studies suggested that IL-6 and STAT3 blockade partially reversed OA damage, mainly by regulating ECM production and degradation [38]. Combining these findings, the IL-6/STAT3 signaling pathway is now reckoned to be a critical target for alleviating cartilage damage.

**Fig. 8:**
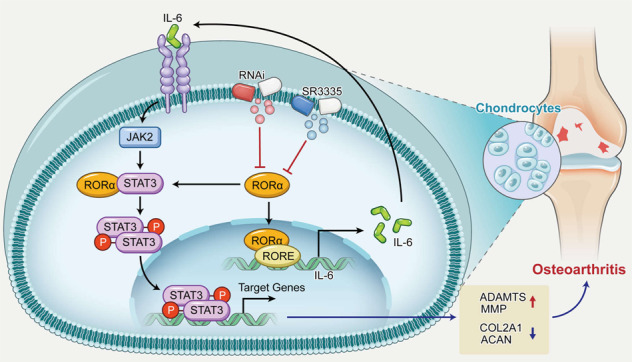
RORα blockade inhibited STAT3 phosphorylation and dampened IL-6/STAT3 pathway in chondrocytes of OA. RORα directly interacts with STAT3 protein or the promoter region of *IL6* gene, thereby accelerating the cartilage depredating effect of inflammatory cytokine. RORα blockade with either SR3335 or small interfering RNA resulted in the decreased level of STAT3 phosphorylation, and subsequently restored the expression of aggrecan, collagen type II, MMP, and ADAMTS. Finally, RORα blockade ameliorated the progression of cartilage damage.

Accumulating evidence suggested that STAT3 and RORα are closely related transcriptional factors, which respond to the stimulation of IL-6 and TGF-β and initiate Th17 differentiation [39, 40]. RORα regulates certain signaling pathways through direct interaction with key factors, such as β-catenin, YAP, and P65 [7, 41, 42]. However, the exact mechanism that STAT3 cooperated with RORα and aggravates their transcriptional function remains to be investigated. Moreover, RORα could regulate the transcription of IL-6, IL-10, and IL-17 in different cell types [43, 44]. Considering the complex function of RORα, we aim to investigate its interaction with both DNA and protein. This study revealed that RORα could regulate the phosphorylation of STAT3, possibly through transcriptional regulation of the IL6 gene and direct binding with STAT3 (Fig. 8).

In conclusion, RORα blockade exerted a cartilage protection effect by affecting matrix catabolism and anabolism. The IL-6/STAT3 pathway was involved in the regulatory role of RORα deeply, suggesting a potential target for alleviating the cartilage damage in OA.

## Materials and methods

### Mouse OA model

A total of 48 female specific-pathogen-free C57BL/6 mice aged 16 weeks old (East Campus SYSU, Guangzhou, China) were used in the experimental phase of this study. The mice were randomly divided into four groups (*n* = 12): SHAM group, ACLT group, ACLT + si-NC group, and ACLT + si-Rora group. ACLT surgery was performed in the latter three groups, whereas the sham group underwent anesthesia and skin and joint capsule incision without transaction of the anterior cruciate ligament (ACL). Routine disinfection was performed before the operation. The mice were anesthetized with an intraperitoneal injection of 30 mg/kg (body weight) pentobarbital and then fixed in the supine position. A median skin incision was made using sterilized surgical instruments. The patella was reflected laterally to expose the ACL and femoral condyles. A vertical incision was made to the anterior cruciate ligament without compromising other ligaments under a stereomicroscope. Then, the wound was closed layer by layer to complete the operation. All animal experiments were approved by the Sun Yat-sen University Animal Ethics Committee (Approval no: SYSU-IACUC-2020-B376).

### Intra-articular injection in OA mice model

Articular injections were performed using a microsyringe with 34 G insulin needle. Two weeks after ACLT, the indicated adenovirus was injected intraarticularly once a week. An amount of 1 × 10^9^ PFU of adenovirus and a volume of 10 μl was injected. Half of the mice were sacrificed 6 weeks after the initial injection, and the rest were sacrificed 12 weeks after injection. The knee was collected and subjected to histological analysis afterward.

### Antibodies and reagents

These antibodies were obtained from Cell Signaling Technology (Danvers, MA, USA): STAT3 (9139), p-STAT3 (Tyr705, 9145), and SOX9 (82630). Anti-RORα primary antibodies (sc-518081) were purchased from Santa Cruz Biotechnology (Santa Cruz, CA, USA). Antibodies against GAPDH (ab8245), COL2A1 (ab188570, ab34712), MMP13 (ab39012, ab237604), ADAMTS4 (ab185722, ab291548), ADAMTS5 (ab41037), and aggrecan (ab3778) were from Abcam (Cambridge, UK). Goat anti-rabbit (SA00001-2) and anti-mouse (SA00001-1) IgG H&L (HRP) secondary antibodies were purchased from Proteintech (Rosemont, IL, USA). Goat anti-rabbit IgG light chain-specific secondary antibodies (A25022) were obtained from Abbkine (Wuhan, China). Rabbit IgG (2729) was purchased from CST. SR3335 (HY-14413), SR1078 (HY-14422), and Stattic (HY-13818) were purchased from MCE (Monmouth, NJ, USA).

### Collection of human articular cartilage

This study was approved by the Medical Ethics Committee of Sun Yat-sen Memorial Hospital (Approval No: 2021-19). All patients signed a consent form for tissue collection. The OA samples were collected from eight patients with OA (four females and four males; mean age: 66.75 ± 10.13 years). Control samples were obtained from eight patients who underwent amputation or malignant bone tumor resection surgery (three females and five males; mean age: 45.75 ± 23.06 years). Tibial plateau and femoral condyle specimens were collected. All patients were classified according to the K-L scoring system pre-operatively, and the specimens were classified according to the Modified Outerbridge classification. The severe OA was defined as grade III or IV in K-L scoring, and mild OA was defined as grade I or II in K-L scoring.

### Isolation and culture of primary articular chondrocytes

Primary articular chondrocytes were separated from the human articular cartilage specimen according to the previous protocol [45]. In brief, the surrounding tissue was dissected under a stereomicroscope (M205FA, Leica, Weztlar, Germany) and the articular cartilage was carefully isolated. Then cartilage tissue was digested with 0.25% trypsin (Thermo Scientific, Waltham, MA, USA) for 30 min, and subsequently digested with 200 U/ml of collagenase type II (Sigma-Aldrich, St. Louis, MO, USA) for 4 h at 37 °C with continuous mixing. The chondrocytes were then seeded and cultured in Dulbecco’s modified Eagle medium/F-12 medium (HyClone) supplemented with 10% fetal bovine serum (Thermo Scientific).

### Cell viability assay

For the cell viability assay, we seeded the cell suspension (100 μL/well) in a 96-well plate and incubate the culture plate in an incubator for 24 h. The indicated treatments were added and incubated for 24 h or 48 h. Next, 10 μL of CCK-8 solution (MCE) was added to each well. Culture plates were placed in the incubator and incubated for 2 h. The absorbance at 450 nm was measured using a Sunrise microplate reader (TECAN, Männedorf, Switzerland).

### Western blot analysis

Cultured cells were washed with ice-cold PBS (Hyclone), and harvested with radioimmunoprecipitation assay (Beyotime, Shanghai, China). An equal amount of protein was loaded with sodium dodecyl sulphate–polyacrylamide gel electrophoresis gel and then transferred to nitrocellulose filter membrane (Millipore, Boston, MA, USA). The membranes were then blocked and incubated with the corresponding primary antibodies. Membranes were then incubated with Horseradish peroxidase (HRP)-conjugated secondary antibodies for 1 h. The membranes were visualized using an electrochemiluminescence kit (Millipore). Visualized images were analyzed using the BIO-RAD imaging system (Bio-Rad, Hercules, CA, USA). Semi-quantitative analyses of the images were conducted using ImageJ.

### Real-time PCR assay

Total RNA was extracted from chondrocytes according to the previous method described [46]. Real-time polymerase chain reaction (RT-PCR) was performed with SYBR qRT-PCR SuperMix (Novoprotein, Shanghai, China) via a Roche LightCycler 480 System (Roche, Basel, Switzerland). Relative gene expression was determined by the 2^−ΔΔCt^ method. Each experiment was replicated three times for biological replicates, and the results shown in the figure represent the average ΔCt value of all experiments. The primer sequences used in this study are listed in Supplementary Table 1.

### Enzyme-linked immunosorbent assay (ELISA)

The IL-6 level in culture supernatants was measured using ELISA kits (Neobioscience, Shenzhen, China) according to the manufacturer’s instructions.

### RNA-seq analysis

To generate mRNA-sequencing data, two groups of chondrocytes with different treatments, vehicle, and SR3335 treated, were analyzed by RNA-sequencing. The RNA was extracted with TRIzol and an amount of 1 µg RNA was used as input material for the RNA sample preparations. Sequencing libraries were generated according to the manufacturer’s recommendations. Differential expression analysis of control and SR3335-treated groups (three biological replicates) was performed using the DESeq2 R package. The resulting *P* values were adjusted using Benjamini and Hochberg’s approach for controlling the false discovery rate. The adjusted *P* value <0.05 for certain genes was regarded as differentially expressed. The GSE accession number for the RNA-seq data is GSE172291.

### IHC staining

Human and mouse cartilage tissues were fixed in 10% paraformaldehyde for 48 h, then decalcified with ethylenediaminetetraacetic acid decalcifying fluid (pH = 7.2, Solarbio) for 2 months. The decalcified tissues were embedded in paraffin and sectioned at 5 μm. IHC staining was performed according to our previous protocol [47].

### Immunoprecipitation

The cells were lysed with IP lysis buffer (Beyotime, Shanghai, China). For each group, input protein was separated and stored at −80 °C for further detection. An equal amount of protein from the samples was incubated with specific antibodies for 2 h and then incubated with protein A/G magnetic beads (MCE) and spun to mix overnight at 4 °C. The agarose-bound protein was washed with wash buffer five times and separated by the magnetic stand.

### ChIP assay

In brief, 1 × 10^7^ cells were cross-linked with 1% (w/v) formaldehyde and incubated for 10 min at room temperature. Glycine was added and incubated for 5 min to quench the formaldehyde. Cells were lysed using lysis buffer and cell slurry was collected. Then, the cold shearing buffer was added and then sheared into small chromatin fragments between 150–300 bp. Anti-RORα or rabbit IgG antibodies were incubated with the sonicated chromatin overnight at 4 °C. After overnight incubation, the antibody-chromatin mixtures were separated by the magnetic stand. The anti-rabbit A/G beads were pre-blocked and incubated on a rotating tube rack or platform at 4 °C for 6 h. After discarding the supernatant, the samples were placed at 65 °C overnight for reverse cross-linking.

### Oligonucleotide and lentivirus transfection for RORα knockdown

The oligonucleotides for knockdown were synthesized and packaged in virus vectors by Genechem (Shanghai, China). Sequences of single-stranded short interfering hairpin RNA and short hairpin RNA constructs are listed in Supplementary Table 2. For in vitro cell experiments, chondrocytes were infected with lentivirus for 72 h, and the transfection efficiency was observed with fluorescence microscopy. Infection was regarded as successful if the transfection efficiency >80%.

### Statistics

All results represent the mean ± standard deviation. Statistical analysis was performed using the two-tailed independent Student’s *t* test for comparisons of two independent groups, two-tailed paired-sample *t* test for comparisons of two matched groups. One-way analysis of variance followed by Dunnett’s test for comparisons of three or more groups. Non-parametric data were compared by Mann–Whitney *U* test. For all statistical analyses, differences with *P* values < 0.05 were considered statistically significant, and experiments were repeated for indicted times in the figure legend. GraphPad Prism 8.0 was purchased from GraphPad Software (San Diego, CA, USA) was used for data analysis and chart presentation.

## Supplementary information

Supplementary Figures and Tables

## Data Availability

All data generated or analyzed during this study are included in this article and its supplementary files.
