# Supplementary material for: Inhibition of nuclear receptor RORα attenuates cartilage damage in osteoarthritis by modulating IL-6/STAT3 pathway
Source: Cell Death Dis. 2021 Sep 28;12(10):886. doi: 10.1038/s41419-021-04170-0 (PMC8478978; doi:10.1038/s41419-021-04170-0)
Supplement: Supplementary file 1 — Supplementary Figures and Tables [file 41419_2021_4170_MOESM1_ESM.pdf]

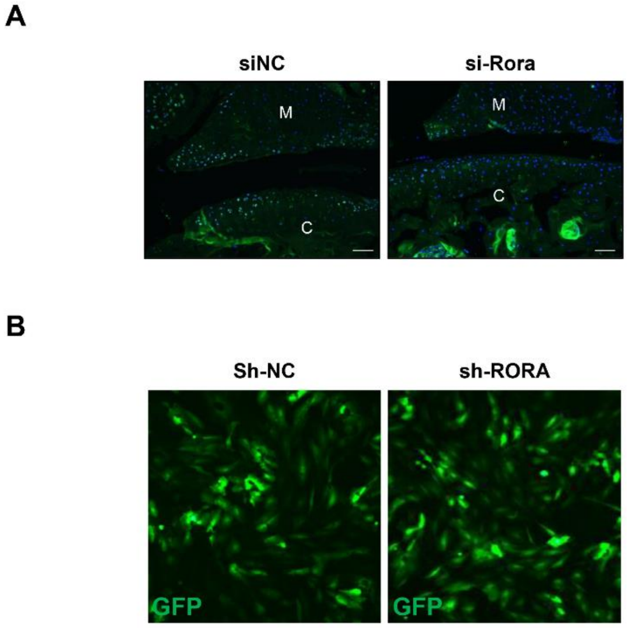

Supplementary Figure 1 The infection efficiency in vivo and in vitro. (A) The immunofluorescence image of RORα (green) and DAPI (blue) 5 days after intra-articular injection of si -Rora or si-NC; M, meniscus; AC, articular cartilage. Scale bars, 100μm. **(B)** Fluorescence of GFP in human chondrocyte infected with sh-RORA or sh-NC lentivirus at 80 MOI separately.

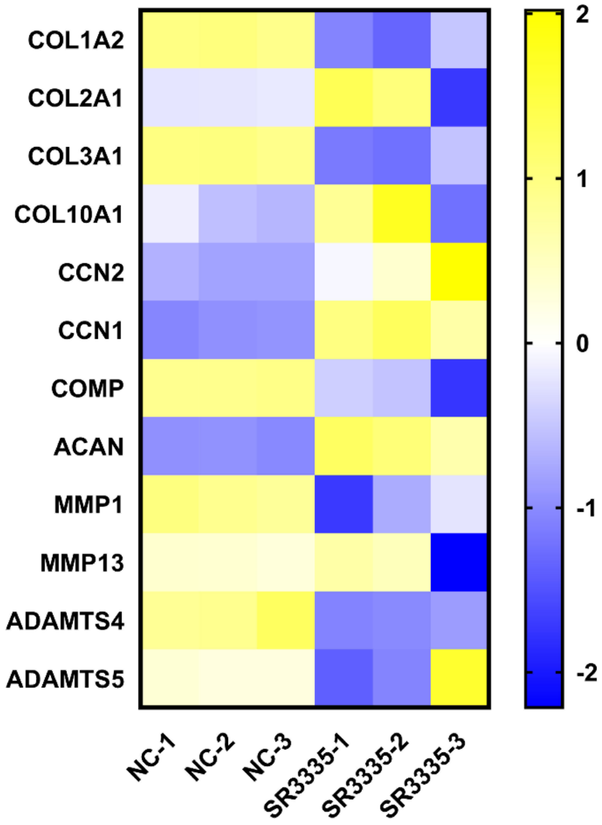

Supplementary Figure 2 The heat map for certain genes after SR3335 treatment.

The relative expression of COL1A2, COL2A1, COL3A1, COL10A1, CCN2, CCN1, COMP, ACAN, MMP1, MMP13, ADAMTS4, and ADAMTS5 was shown.

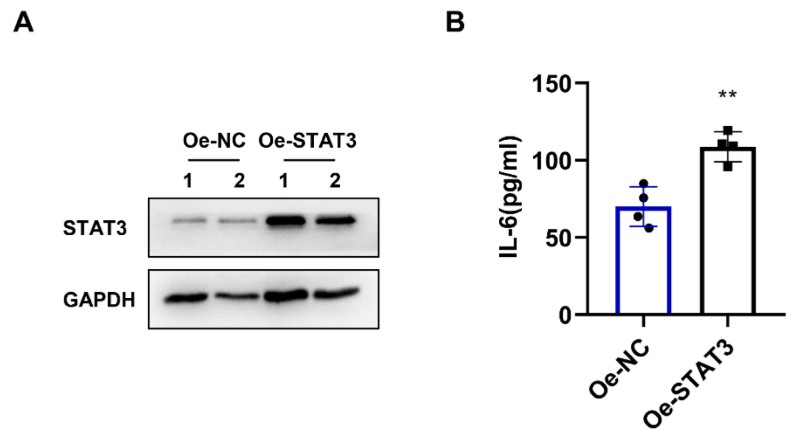

Supplementary Figure 3 The concentration of IL-6 after overexpressing STAT3. **(A)** The chondrocytes were transfected with STAT3 overexpression plasmid and control plasmid for 48 h. The expression level of STAT3 protein was detected with Western blot. **(B)** ELISA assay to detect IL-6 protein expression after transfected with STAT3 overexpression or control plasmid for 48 h. n = 4 independent experiments. Data in (B) are presented as mean ± SD. The statistical data in (B) was analyzed with Student's t test. ** $P < 0.01$. All data shown above are presented as mean ± SD.

Supplementary Table 1 Primer sequence used in this study

Gene	Forward/ Reverse	Primer sequence (5′-3′)
RORA	Forward	ACTCCTGTCCTCGTCAGAAGA
	Reverse	CATCCCTACGGCAAGGCATTT
COL2A1	Forward	GGCAATAGCAGGTTCACGTACA
	Reverse	CGATAACAGTCTTGCCCCACTT
ACAN	Forward	TGCATTCCACGAAGCTAACCTT
	Reverse	GACGCCTCGCCTTCTTGAA
COL10A1	Forward	CAAGGCACCATCTCCAGGAA
	Reverse	AAAGGGTATTTGTGGCAGCATATT
SOX9	Forward	AGCGAACGCACATCAAGAC
	Reverse	GCTGTAGTGTGGGAGGTTGAA
MMP13	Forward	CCAGACTTCACGATGGCATTG
	Reverse	GGCATCTCCTCCATAATTTGGC
ADAMTS4	Forward	GAGGAGGAGATCGTGTTTCCA
	Reverse	CCAGCTCTAGTAGCAGCGTC
ADAMTS5	Forward	GAACATCGACCAACTCTACTCCG
	Reverse	CAATGCCCACCGAACCATCT
IL6	Forward	ACTCACCTCTTCAGAACGAATTG
	Reverse	CCATCTTTGGAAGGTTCAGGTTG
IL6R	Forward	CCCCTCAGCAATGTTGTTTGT
	Reverse	CTCCGGGACTGCTAACTGG
STAT3	Forward	CAGCAGCTTGACACACGGTA
	Reverse	AAACACCAAAGTGGCATGTGA
SOCS3	Forward	CCTGCGCCTCAAGACCTTC
	Reverse	GTCACTGCGCTCCAGTAGAA
JAK2	Forward	TCTGGGGAGTATGTTGCAGAA
	Reverse	AGACATGGTTGGGTGGATACC
IL1B	Forward	AGCTACGAATCTCCGACCAC
	Reverse	CGTTATCCCATGTGTCGAAGAA
IL17A	Forward	TCCCACGAAATCCAGGATGC
	Reverse	GGATGTTCAGGTTGACCATCAC
TNF	Forward	CCTCTCTCTAATCAGCCCTCTG
	Reverse	GAGGACCTGGGAGTAGATGAG
GAPDH	Forward	AGAAAAACCTGCCAAATATGATGAC

	Reverse	TGGGTGTCGCTGTTGAAGTC

Supplementary Table 2 Oligo sequence used in this study

Oligo	Forward/ Reverse	Primer sequence (5′-3′)
Si-Rora-1	Forward	CCGGGCGCAGGCAGAGCTATGCGAGCTCG AGCTCGCATAGCTCTGCCTGCGCTTTTTG
	Reverse	AATTCAAAAAGCGCAGGCAGAGCTATGCG AGCTCGAGCTCGCATAGCTCTGCCTGCGC
Si-Rora-2	Forward	CCGGCAGGCAGAGCTATGCGAGCTCCTCG AGGAGCTCGCATAGCTCTGCCTGTTTTTG
	Reverse	AATTCAAAAACAGGCAGAGCTATGCGAGC TCCTCGAGGAGCTCGCATAGCTCTGCCTG
Si-Rora-3	Forward	CCGGGCTATGCGAGCTCCAGCCGAGCTCG AGCTCGGCTGGAGCTCGCATAGCTTTTTG
	Reverse	AATTCAAAAAGCTATGCGAGCTCCAGCCG AGCTCGAGCTCGGCTGGAGCTCGCATAGC
Sh-RORA-1	/	GGAGAAGTCAGCAAAGCAATGCTCGAGC ATTGCTTTGCTGACTTCTCC
Sh-RORA-2	/	GCAGAGAGACAGCTTGTATGCCTCGAGGC ATACAAGCTGTCTCTCTGC
Sh-RORA-3	/	AAACGTGACACGTTCGGAGAACGAATTCT CCGAACGTGTCACGTTT
